# Transitory restless arms syndrome in a patient with antipsychotics and antidepressants: a case report

**DOI:** 10.1186/s12888-021-03433-6

**Published:** 2021-09-16

**Authors:** Juan Chen, Na Meng, Bingrong Cao, Yinghua Ye, Ying Ou, Zhe Li

**Affiliations:** 1 Mental Health Center, West China Hospital/West China School of Nursing, Sichuan University, Chengdu, China; 2 Mental Health Center, West China Hospital, Sichuan University, No.28 Dianxin South Road, Chengdu, 610041 China; 3 Sichuan Clinical Medical Research Center for Mental Disorders, Chengdu, China

**Keywords:** Restless arms syndrome, Antipsychotics, Antidepressants, Case report

## Abstract

**Background:**

Restless arms syndrome (RAS) is characterized by uncomfortable aching or burning sensations in the arms. RAS is regarded as an upper limb variant of restless legs syndrome (RLS). The lack of specific diagnostic criteria makes it difficult to recognize the RAS. Therefore, RAS is usually neglected in clinical practice. Moreover, when a patient was diagnosed with RAS, the adjustment of medications was the first choice for doctors, which may make the patient’s condition unstable.

**Case presentation:**

A 33-year-old woman was diagnosed with schizophrenia and major depressive disorder. Starting with 0.6 g/d amisulpride, 0.1 g/d quetiapine, 75 mg/d venlafaxine sustained-release tablets, the patient reported symptoms of RAS (itching arms) on the fourth day since the latest hospitalization. After ruling out other factors, her RAS was suspected to be induced by antidepressants or antipsychotics. Without medication adjustment, RAS spontaneously remitted.

**Conclusions:**

This case suggests that psychiatrists should pay attention to RAS when using antipsychotics and/or antidepressants. Moreover, RAS may be transitory. When a patient manifests RAS, observation may be one choice instead of an immediate medication adjustment.

## Background

Restless arms syndrome (RAS) is usually defined by upper limbs restlessness [1]. It is characterized by uncomfortable aching or burning sensations in the arms and an urge to move arms [1]. Those unpleasant sensations and restlessness have a negative effect on sleep [2] and even lead to suicidal thoughts [3]. RAS may be related to antipsychotics [4] and could be released by dopaminergic treatment [5] as well as complementation of iron [2]. However, a previous review showed that RAS might be an extremely rare disease with unknown prevalence and no specific diagnostic criteria [1]. Generally, RAS is regarded as an upper limb variant of Restless legs syndrome (RLS) [6]. Arm restlessness, as a late feature, was reported by nearly half of the patients diagnosed with RLS [7]. However, the lack of specific diagnostic criteria makes it difficult to recognize RAS. Therefore, RAS is usually neglected in clinical practice. In addition, the relationship between RAS and antidepressants as well as antipsychotics remains unknown. Moreover, when a patient was diagnosed with RAS, the adjustment of medication was the first choice for doctors in previous reports [1]. However, changes in medication may make the patient’s condition unstable. Here, we report a rare case of RAS that may be induced by antidepressants and/or antipsychotics. More importantly, we first reported a case of RAS with spontaneous remission without medication adjustment, which may help us to better understand and deal with RAS.

## Case presentation

**Fig. 1:**
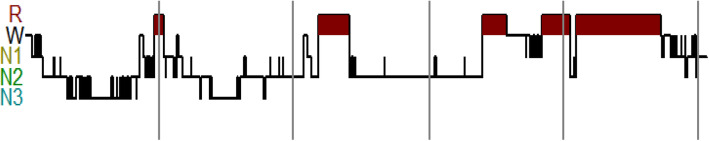
The Hypnogram of the case. Abbreviations: R, rapid eye movement; N1, the first stage of nonrapid eye movement; W, wakeup; N2, the second stage of nonrapid eye movement; N3, the third stage of nonrapid eye movement

The patient was a female aged 33 years old. Three years ago, she manifested auditory hallucinations (hearing that someone was talking about her), persecutory delusions (feeling that someone would kill her) and disorganized behaviour (talking solely, laughing solely, and saying that she was a robot). She was diagnosed with schizophrenia by The Diagnostic and Statistical Manual of Mental Disorders, 5th Edition (DSM-5) [8]. After a short-term hospitalization, she went home with symptom remission. The treatment method was not clear. Although she did not take medications regularly after discharge, she could work and live normally. One year ago, similar symptoms recurred. She was discharged with remission of symptoms by 0.2 g/d quetiapine and 7.5 mg/d dexzopiclone. Three months ago, she felt stressed and worried that someone would kill her again. After prescribing amisulpride, quetiapine, benzhexol hydrochloride and propranolol hydrochloride, the symptoms were controlled. The exact doses were not clear. After discharge, she took those medications regularly. Ten days ago, she was depressed because she could not get a job. She felt useless and complained of palpitations. She also said that a robot appeared around her. She was diagnosed with schizophrenia and major depressive disorder by DSM-5 [8]. Except for increased prolactin (195.7 ng/ml; reference range, 6.0–29.9 ng/ml) and cortisol (659.4 nmol/l; reference range, 147.3–609.3 nmol/l), blood examination showed no abnormalities. Ferritin (88.30 ng/ml; reference range 24–336 ng/ml) and renal function (glomerular filtration rate = 104.47 ml/min/1.73m^2^; reference range, 56–122 ml/min/1.73m^2^) were in the normal range. She had no pregnancy and immune diseases. Physical examination and imaging diagnosis showed no abnormalities. She also had no family history of psychiatric or neurological illness. Treatment was started with 0.6 g/d amisulpride to control psychiatric symptoms, 0.1 g/d quetiapine to improve sleep, 75 mg/d venlafaxine sustained-release tablets to relieve depression, 4 mg/d benzhexol hydrochloride and 20 mg/d propranolol hydrochloride. After 3 days, the patient reported daytime sleepiness and itch in her arms. When she rested, the itch worsened. In contrast, the itch could be relieved when she moved her arms. There was no rash, redness, swelling or pain on the arms. She did not report any discomfort in the legs. A nocturnal polysomnogram but not electromyogram channels in the arms was conducted on the patient. The time of wakefulness were 47.5 min. The numbers of wakefulness were 14. The periodic limb movement index was 6. The results indicated poor sleep continuity (see Table 1 and Fig. 1 in detail). She was diagnosed with RAS, a variant of RLS, according to the International RLS Study Group Criteria [9]. On the second day, the patient reported remission of itch in her arms without adjusting the medication. She did not complain of itch on the arms again in the ward and the half-year follow-up period.

**Table 1:** The results of nocturnal polysomnogram

Index	Results
Time in bed, min	608
Total sleep time, min	553.5
Numbers of wakefulness	14
Time of wakefulness, min	47.5
NREM 1, min (%)	69.0 (12.5%)
NREM 2, min (%)	259.5 (46.9%)
NREM 3, min (%)	66.0 (11.9%)
REM, min (%)	159.0 (28.7%)
Arousal index, No./h	8.4
Sleep onset latency, min	7.0
REM latency, min	108.5
Respiratory disturbance index, No./h	1.3
Lowest arterial oxygen saturation, %	92
Limb movement index, No./h	9.1
Periodic limb movement index, No./h	6.0

Abbreviations: NREM, nonrapid eye movement; REM, rapid eye movement

## Discussion and conclusions

Although there is no diagnostic criterion specific for RAS, similar diagnostic criteria for RLS exist. RAS is regarded as an RLS variant [1]. The patient with RAS manifested paresthesias in the upper extremities. The arms were affected predominantly, and legs were affected little or not involved. Therefore, RAS is difficult to diagnose and may be easily ignored by physicians. This case reinforced physicians could consider the possibility of RAS when using antidepressants and/or antipsychotics.

The patient was, at the time of presentation, taking two antipsychotic medications, which could result in the side effect of akathisia which is a form of restlessness. However, the symptoms of akathisia could not improve by the movement of upper limbs [10]. In our case, when she rested, the itch worsened. In contrast, the itch could be relieved when she moved her arms. Moreover, sleep disturbance would not usually present in a patient with akathisia [10].

The patient’s main complaint was transitory itchiness, which has many potential causes. One possibility is a reaction to the sleeves of a garment, laundered perhaps with allergenic soap. However, the patient’s itchiness relieved from the movement of upper limbs. There was no rash, redness, swelling or pain on the arms. Therefore, the patient is more likely to be diagnosed with RAS.

Another interesting finding is that the itching symptom disappeared without any medication discontinuation or intervention. The result differentiates from previous cases in RLS. Previous studies reported that leg symptoms were usually remitted after the withdrawal of antidepressants or quetiapine [11]. There is a titration process in the treatment of antidepression or antipsychotics. The medication adjustment resulting from RAS would increase the period of medication therapy from the initial dose to the maintenance dose. The nocturnal polysomnogram indicated normal periodic limb movement in the patient, and the obvious arm symptoms disappeared spontaneously without any intervention. Therefore, we did not address RAS or periodic limb movement. Our case suggests that RLS may only feature arm symptoms. In addition, RAS may be spontaneous remission without any intervention.

RAS may result from iron deficiency, pregnancy, end-stage renal disease and rheumatic diseases [1]. In our case, ferritin and glomerular filtration rate were normal, which suggested that the patient have no iron deficiency or renal diseases. In addition, the patient also had no pregnancy or immune diseases. Therefore, the possible reason for RAS is the use of medications. However, medication-induced RAS is seldomly reported. Moreover, RAS is regarded as the variant of RLS. In fact, there is no overwhelming evidence of why RLS happens in patients with antidepressants or antipsychotics. The sort of medications may be risk factors for RLS. RLS might be induced by a single antidepressant or antipsychotic. Several studies thought that RLS was a side effect of quetiapine [12]. When the patient was under monotherapy, the most common reason for RLS was quetiapine (28.5%) in the antipsychotic group and paroxetine (22.2%) in the antidepressant group [13]. However, a low concentration of amisulpride may improve RLS resulting from dopaminergic agonist effects in the spinal area [14]. Another case pointed out that RLS symptoms improved rapidly on 0.5 mg of clonazepam and 50 mg of quetiapine after adjustment from 1 mg of clonazepam [15]. In addition, combined medications may increase the risk of RLS. Ocak [16] reported that patients with anxiety or depression who received combined drug treatment, such as selective serotonin reuptake inhibitors (SSRIs) + quetiapine and SSRIs + mirtazapine, had a 4.7-fold increase in RLS. In our case, the patients used three medications, including amisulpride, quetiapine and venlafaxine sustained-release tablets. As a result of not withdrawing medication, we cannot be sure which medication induced or improved the RAS in our case.

The dose of quetiapine and combined antidepressant medications may have an impact on the occurrence of RAS. One report showed that secondary RLS was a dose-dependent side effect of quetiapine [17]. A previous study revealed that quetiapine at doses of 200–600 mg/d induced RLS [18]. Another case pointed out that quetiapine at a low dose (50 mg/d) also resulted in RLS in a 75-year-old woman with insomnia [19]. Furthermore, RLS presented at very small to moderate doses (25–250 mg) in patients with affective disorders on treatment with antidepressants and quetiapine [20]. Similar to previous conclusions, the dose of quetiapine (100 mg/d) was also small in our case, which may induce RAS.

The possible mechanisms between RAS and antidepressants or antipsychotics in our patient may be as follows. First, central dopaminergic dysfunction may play an important role in the relationship between quetiapine and RLS. RLS was induced by antagonize dopamine receptors [21]. Although the D2 receptor affinity ratio of quetiapine and amisulpride was lower than that of other antipsychotic medications, such as olanzapine and ziprasidone, a sufficient ratio of initial transient binding with D2 receptors might be the reason for RLS [22]. On the other hand, RLS induced by quetiapine might be associated with 5-hydroxytryptamine (5-HT) agonism. Quetiapine has a strong antagonistic effect on 5-HT, which can increase the inhibitory effect of dopaminergic neurotransmission by 5-HT [23]. Moreover, quetiapine, with a high affinity for histamine1 receptors, presents antihistamine action that is thought to be related to the occurrence of RLS [24]. Venlafaxine hydrochloride may lead to dopaminergic hypoactivity resulting from enhanced serotonin-mediated inhibition of dopaminergic neurotransmission [25]. In addition, the occurrence of antipsychotic-induced RLS may result from cytochrome P450 enzymes. Cytochrome P450 enzymes are related to the plasma levels of antipsychotics and antidepressants [26]. A study pointed out that the value of O-desmethylvenlafaxine, an active metabolite of venlafaxine, is higher when quetiapine is coadministered [27]. A possible reason is that CYP3A4, a sort of cytochrome P450, is the main metabolizing enzyme for quetiapine as well as the metabolizing enzyme for venlafaxine [27]. Quetiapine reduces the metabolism of venlafaxine via CYP3A4, which may lead to the side effects of RAS. Amisulpride is directly absorbed by the gastrointestinal tract and eliminated by the kidneys as an unchanged drug [28]. Therefore, amisulpride has no effect on the liver metabolizing process of quetiapine and venlafaxine. Gender may be another influencing factor of RAS. In a nationwide survey, Tison et al. [29] reported that 10% of women and 5% of men had RLS in 10,263 French adults. Another study also found that females accounted for 69% of patients with RLS [30]. In our case, the patient was female. The case pointed out that females may have a higher likelihood of suffering from RAS.

In conclusion, RAS may be present in an individual with a small to moderate dose of quetiapine or combined with antidepressants. Psychiatrists should also be aware of RAS, especially in female patients with schizophrenia. Polysomnograms could be beneficial for the diagnosis of RAS. More importantly, the case pointed out that the RAS may be transient and does not have a continuous effect on patients. Psychiatrists may not have to immediately change the medication regimen when the RAS appears. When a patient manifests RAS, observation may be a choice instead of an immediate medication adjustment. Moreover, further studies should explore the underlying mechanisms of RAS and its management strategies.

## Data Availability

This is a single-patient case report. Data sharing is not applicable to this article as no datasets besides those mentioned in the article were generated or analyzed.

## Abbreviations

RAS: Restless arms syndrome
RLS: Restless legs syndrome
DSM-5: The Diagnostic and Statistical Manual of Mental Disorders, 5th Edition
